# A Note on the Conditioning of the **H**^−1^ Matrix Used in Single-Step GBLUP

**DOI:** 10.3390/ani12223208

**Published:** 2022-11-19

**Authors:** Mohammad Ali Nilforooshan

**Affiliations:** Livestock Improvement Corporation, Private Bag 3016, Hamilton 3240, New Zealand; mohammad.nilforooshan@lic.co.nz

**Keywords:** conditional property, inflated, relationship matrix, single-step GBLUP, weighting

## Abstract

**Simple Summary:**

Compared to BLUP, in single-step genomic BLUP, $G^{-1}-A_{22}^{-1}$ is added to the inverse of the pedigree relationship matrix ($A^{-1}$), forming $H^{-1}$, where **G** is the genomic relationship matrix, and $A_{22}$ is the block of **A** for genotyped animals. Incompatibility between **G** and **A** may cause inflated genetic variance. Blending and tuning **G** with $A_{22}$ partially solves the problem. However, conditioning $H^{-1}$ might still be needed, which is usually performed via $\tau G^{-1}-\omega A_{22}^{-1}$. This may violate the properties upon which **H** is built. Alternative ways of weighting the $H^{-1}$ components are presented to prevent/minimise violations of the properties of **H**.

**Abstract:**

The single-step genomic BLUP (ssGBLUP) is used worldwide for the simultaneous genetic evaluation of genotyped and non-genotyped animals. It is easily extendible to all BLUP models by replacing the pedigree-based additive genetic relationship matrix (**A**) with an augmented pedigree–genomic relationship matrix (**H**). Theoretically, **H** does not introduce any artificially inflated variance. However, inflated genetic variances have been observed due to the incomparability between the genomic relationship matrix (**G**) and **A** used in **H**. Usually, **G** is blended and tuned with $A_{22}$ (the block of **A** for genotyped animals) to improve its numerical condition and compatibility. If deflation/inflation is still needed, a common approach is weighting $G^{-1}-A_{22}^{-1}$ in the form of $\tau G^{-1}-\omega A_{22}^{-1}$, added to $A^{-1}$ to form $H^{-1}$. In some situations, this can violate the conditional properties upon which **H** is built. Different ways of weighting the $H^{-1}$ components ($A^{-1}$, $G^{-1}$, $A_{22}^{-1}$, and $H^{-1}$ itself) were studied to avoid/minimise the violations of the conditional properties of **H**. Data were simulated on ten populations and twenty generations. Responses to weighting different components of $H^{-1}$ were measured in terms of the regression of phenotypes on the estimated breeding values (the lower the slope, the higher the inflation) and the correlation between phenotypes and the estimated breeding values (predictive ability). Increasing the weight on $H^{-1}$ increased the inflation. The responses to weighting $G^{-1}$ were similar to those for $H^{-1}$. Increasing the weight on $A^{-1}$ (together with $A_{22}^{-1}$) was not influential and slightly increased the inflation. Predictive ability is a direct function of the slope of the regression line and followed similar trends. Responses to weighting $G^{-1}-A_{22}^{-1}$ depend on the inflation/deflation of evaluations from $A^{-1}$ to $H^{-1}$ and the compatibility of the two matrices with the heritability used in the model. One possibility is a combination of weighting $G^{-1}-A_{22}^{-1}$ and weighting $H^{-1}$. Given recent advances in ssGBLUP, conditioning $H^{-1}$ might become an interim solution from the past and then not be needed in the future.

## 1. Introduction

The unified genetic evaluation of genotyped and non-genotyped animals has been of great interest. In an initial attempt, Misztal et al. [1] suggested a unified pedigree (**A**) and genomic (**G**) relationship matrix ($H_{\mathrm{ini}}$), in which genomic relationships between genotyped animals replaced their pedigree relationship coefficients in **A**. Denoting non-genotyped and genotyped animals with 1 and 2:

(1) $$H_{\mathrm{ini}}=\left[\begin{array}{cc} A_{11} & A_{12}\\ A_{21} & G \end{array}\right]=A+\left[\begin{array}{cc} 0 & 0\\ 0 & G-A_{22} \end{array}\right].$$

This relationship matrix did not condition the distributions of breeding values for genotyped and non-genotyped animals on each other, leading to incoherencies in the joint distribution of genetic values for genotyped and non-genotyped animals. Legarra et al. [2] presented an augmented (**A** and **G**) relationship matrix in which the genetic values of non-genotyped animals were conditioned to the genetic values of genotyped animals. The resulting matrix was:

(2) $$\begin{array}{c} \begin{array}{cc} H & =\left[\begin{array}{cc} H_{11} & H_{12}\\ H_{21} & H_{22} \end{array}\right]\\ & =\left[\begin{array}{cc} A_{11}+A_{12}A_{22}^{-1}\left(G-A_{22}\right)A_{22}^{-1}A_{21} & A_{12}A_{22}^{-1}G\\ GA_{22}^{-1}A_{21} & G \end{array}\right]\\ & =A+\left[\begin{array}{cc} A_{12}A_{22}^{-1}\left(G-A_{22}\right)A_{22}^{-1}A_{21} & A_{12}A_{22}^{-1}\left(G-A_{22}\right)\\ \left(G-A_{22}\right)A_{22}^{-1}A_{21} & G-A_{22} \end{array}\right], \end{array} \end{array}$$

which can be simplified to any of the following:

(3) $$\begin{array}{cc} H & =\left[\begin{array}{cc} A_{11}-A_{12}A_{22}^{-1}A_{21} & 0\\ 0 & 0 \end{array}\right]+\left[\begin{array}{c} A_{12}A_{22}^{-1}\\ I \end{array}\right]G\left[\begin{array}{c} A_{22}^{-1}A_{21}\\ I \end{array}\right], \end{array}$$

(4) $$\begin{array}{cc} H & =\left[\begin{array}{cc} {\left(A^{11}\right)}^{-1} & 0\\ 0 & 0 \end{array}\right]+\left[\begin{array}{c} A_{12}A_{22}^{-1}\\ I \end{array}\right]G\left[\begin{array}{c} A_{22}^{-1}A_{21}\\ I \end{array}\right], \end{array}$$

(5) $$\begin{array}{cc} H & =A+\left[\begin{array}{c} A_{12}A_{22}^{-1}\\ I \end{array}\right]\left(G-A_{22}\right)\left[\begin{array}{c} A_{22}^{-1}A_{21}\\ I \end{array}\right]. \end{array}$$

In matrix **H**, the genomic information in **G** influences the relationships between non-genotyped and genotyped animals and among non-genotyped animals. Later, it was discovered that $H^{-1}$ can be indirectly obtained without forming and inverting **H** [3,4].

(6) $$H^{-1}=\left[\begin{array}{cc} H^{11} & H^{12}\\ H^{21} & H^{22} \end{array}\right]=\left[\begin{array}{cc} A^{11} & A^{12}\\ A^{21} & H^{22} \end{array}\right]=A^{-1}+\left[\begin{array}{cc} 0 & 0\\ 0 & G^{-1}-A_{22}^{-1} \end{array}\right].$$

Note that:

$$\begin{array}{cc} G^{-1}-A_{22}^{-1}\; & =H_{22}^{-1}-A_{22}^{-1}\\ \; & =H^{22}-H^{21}{\left(H^{11}\right)}^{-1}H^{12}-A^{22}+A^{21}{\left(A^{11}\right)}^{-1}A^{12}\\ \; & =H^{22}-A^{21}{\left(A^{11}\right)}^{-1}A^{12}-A^{22}+A^{21}{\left(A^{11}\right)}^{-1}A^{12}\\ \; & =H^{22}-A^{22}. \end{array}$$

Matrix **G** is not always full-rank (e.g., when the number of genotyped animals is greater than the number of loci or when there are duplicated genotypes, such as for identical twins). To force **G** to be positive-definite and avoid large diagonal values of $G^{-1}$ due to the bad numerical condition of **G**, the first step of conditioning **G** often involves blending it with $A_{22}$, which is always positive-definite (except in the existence of identical twins or clones [5]) and of good numerical conditions (i.e., $G\leftarrow \left(1-k\right)G+kA_{22}$, 0 < *k* < 1). Blending introduces residual polygenic effects (genetic effects not captured by genetic markers) to the evaluation model without explicitly modelling it, where the scalar *k* is the ratio of the polygenic to the total additive genetic variance [6].

It is theoretically true that no artificially inflated variance is introduced via the **H** matrix [2]. However, inflated genetic variances have been observed due to incompatibilities between **G** and $A_{22}$ [6,7,8,9]. Incompatible **G** and $A_{22}$ lead to incorrectly weighted pedigree and genomic information [7,8]. Besides different distributions of **G** and $A_{22}$ elements, incomplete and incorrect pedigree information, and genotyping and imputation errors, incompatibilities between **G** and $A_{22}$ can be due to the non-random selection of genotyped animals [10], and the different bases and scales of the two matrices [7]. Matrices $A_{22}$ and **G** regress data to different means. Matrix $A_{22}$ regresses solutions towards pedigree founders, animals in the pedigree with unknown parents or genetic groups if considered in the pedigree. On the other hand, **G** regresses solutions toward a founder population comprising genotyped animals [5,10] since the real allele frequencies in the founder population are unknown. The average genetic merit of genotyped animals can be different from founders, especially in the presence of selection. Different approaches (referred to as tuning) have been used for correcting the base difference between **G** and $A_{22}$ [7,11] and rebasing and scaling **G** to improve its consistency with $A_{22}$ [10]. Those approaches were tested by Nilforooshan [9] on New Zealand Romney sheep. Christensen [8] and Gao et al. [6] tuned **G** by regressing its averages to the averages of $A_{22}$ (Equations (7) and (8), respectively).

(7) $$\begin{array}{cc} \; & \left\{\begin{array}{c} \mu \left(\mathrm{diag}\left(G\right)\right)\beta +\alpha =\mu \left(\mathrm{diag}\left(A_{22}\right)\right)\\ \mu \left(G\right)\beta +\alpha =\mu \left(A_{22}\right) \end{array}\right. \end{array}$$

(8) $$\begin{array}{cc} \; & \left\{\begin{array}{c} \mu \left(\mathrm{diag}\left(G\right)\right)\beta +\alpha =\mu \left(\mathrm{diag}\left(A_{22}\right)\right)\\ \mu \left(\mathrm{offdiag}\left(G\right)\right)\beta +\alpha =\mu \left(\mathrm{offdiag}\left(A_{22}\right)\right) \end{array}\right. \end{array}$$

The α and β scalars obtained by solving either of the equations above are used for transforming **G** into $\beta G+\alpha 11^{\prime }$. Another solution proposed to tackle the problem of inflated genomic evaluations (i.e., an increased variance of genomic predictions) as a result of incorrectly scaled genomic and pedigree information was scaling $G^{-1}-A_{22}^{-1}$ in the form of $\tau G^{-1}-\omega A_{22}^{-1}$ [3,12,13]. Applying $\tau G^{-1}-\omega A_{22}^{-1}$ is equivalent to transforming **G** into ${\left[\tau G^{-1}-\left(\omega -1\right)A_{22}^{-1}\right]}^{-1}$ [3,9], which equals $G{\left[\left(1-\omega \right)G+\tau A_{22}\right]}^{-1}A_{22}$. It is also equivalent to replacing $G-A_{22}$ with ${\left[\tau G^{-1}-\left(\omega -1\right)A_{22}^{-1}\right]}^{-1}$ in Equation (2) [12].

Reducing τ and ω values toward 0 brings **G** closer to $A_{22}$ by bringing $H^{22}$ closer to $A^{22}$. However, it is not easily quantifiable how **G** and $A_{22}$ are proportionally combined. With τ and ω deviating from each other and 1, there is a risk of distorting the conditional properties of **H**, because the changes made in $H^{22}$ are not reflected in other blocks of $H^{-1}$. Whereas 1 – *k* and *k* are the commonly used blending coefficients of **G** and $A_{22}$, τ and ω are the commonly used blending coefficients of $H^{-1}$ and $A^{-1}$. i.e.,

(9) $$A^{-1}+\left[\begin{array}{cc} 0 & 0\\ 0 & \tau G^{-1}-\omega A_{22}^{-1} \end{array}\right]=\omega H^{-1}+\left(1-\omega \right)A^{-1}+\left[\begin{array}{cc} 0 & 0\\ 0 & \left(\tau -\omega \right)G^{-1} \end{array}\right].$$

Considering the above equation, there is no legitimate reason for ω being out of the boundary of 0 and 1, and τ−ω being out of the boundary of –1 and 1. Martini et al. [12] studied τ ranging from 0.1 to 2, and ω ranging from –1 to 1 by steps of 0.1, leading to 420 analyses. Dealing with two parameters increases the number of analyses and validation tests in a two-dimensional space. It is assuming that the *k* coefficient has already been chosen and does not need to be validated. The most coherent approach for finding *k* is by restricted maximum likelihood (REML), as proposed by Christensen and Lund [4], rather than using empirical values by screening and validation.

Weighting $G^{-1}$ and $A_{22}^{-1}$ as $\tau G^{-1}-\omega A_{22}^{-1}$ has been used until recently [12,13,14,15,16,17]. Several improvements have been made to ssGBLUP [18] and the use of $\tau G^{-1}-\omega A_{22}^{-1}$ is declining. For example, one of the factors leading to the need for an ω considerably less than 1 was that inbreeding coefficients were considered in $A_{22}^{-1}$ but not in $A^{-1}$ [19]. The aim of this study was to communicate the problems that might occur using $\tau G^{-1}-\omega A_{22}^{-1}$, and investigate the possible solutions for weighting the $H^{-1}$ components if the modifications in **G** are not satisfactory and the weighting of the $H^{-1}$ components is still needed for the deflation/inflation of genomic breeding values.

## 2. Methods

### 2.1. Possible Problems with $\tau G^{-1}-\omega A_{22}^{-1}$

The $\left(\tau -\omega \right)G^{-1}$ matrix in Equation (9) is unconditional and not reflected in the other blocks of $H^{-1}$. As such, some combinations of τ≠ω potentially distort the conditional properties of **H**. However, any τ=ω ranging from 0 to 1 is legitimate and can be considered as a blending of $H^{-1}$ and $A^{-1}$. While it might make sense to weight $G^{-1}$ and $A_{22}^{-1}$ to bring them closer to each other and make them more compatible, weighting $A_{22}^{-1}$ causes incompatibility between $A_{22}^{-1}$ and $A^{-1}$. Matrix $H^{-1}$ can also be written as:

(10) $$\begin{array}{cc} H^{-1}\; & =\left[\begin{array}{c} I\\ -A_{22}^{-1}A_{21} \end{array}\right]A^{11}\left[\begin{array}{c} I\\ -A_{12}A_{22}^{-1} \end{array}\right]+\left[\begin{array}{cc} 0 & 0\\ 0 & G^{-1} \end{array}\right] \end{array}$$

(11) $$\begin{array}{cc} \; & =\left[\begin{array}{cc} A^{11} & A^{12}\\ A^{21} & A^{22}-A_{22}^{-1} \end{array}\right]+\left[\begin{array}{cc} 0 & 0\\ 0 & G^{-1} \end{array}\right]. \end{array}$$

Weighting the components of $\left[\begin{array}{c} I\\ -A_{22}^{-1}A_{21} \end{array}\right]A^{11}\left[\begin{array}{c} I\\ -A_{12}A_{22}^{-1} \end{array}\right]$ in Equation (10), the aim is to preserve the existing quadratic form. This study aimed to introduce weighting on the $H^{-1}$ components that are unlikely to introduce distortions to the conditional properties of **H**. Weighting $H^{-1}$ can be performed on any of the following components:

1. $H^{-1}$ itself
2. $G^{-1}-A_{22}^{-1}$
3. $G^{-1}$
4. $A^{-1}$
5. $A^{11}$
6. $A^{22}$
7. $A_{22}^{-1}$

### 2.2. Weighting $H^{-1}$

This scenario is helpful when the heritability estimate (h^2^) does not match the data or $H^{-1}$. Heritability may change over time and as a result of selection. An outdated h^2^ may differ from the current h^2^ of the trait in the population. Estimating variance components is a computationally expensive process. The h^2^ estimate might have been from a population subset or via a matrix other than $H^{-1}$ ($A^{-1}$ or $G^{-1}$). Different relationship matrices contain different information and may result in different genetic variances and h^2^ estimates [20].

### 2.3. Weighting $G^{-1}-A_{22}^{-1}$

Aguilar et al. [3] suggested using equal τ and ω. Weighting $G^{-1}-A_{22}^{-1}$ by α is equivalent to $\alpha H^{-1}+\left(1-\alpha \right)A^{-1}$.

### 2.4. Weighting $G^{-1}$

This scenario can be understood as scaling the h^2^ corresponding to $G^{-1}$ to the h^2^ corresponding to $A^{-1}$. No violation is made to the conditional properties of $H^{-1}$, and weighting $G^{-1}$ by α is equivalent to using G/α in **H**. Therefore, instead of **G**, G/α is propagated through the blocks of **H**. A G/α more compatible with $A_{22}$ would bring **G** closer to and more compatible with **A**.

### 2.5. Weighting $A^{-1}$

This scenario can be understood as scaling the h^2^ corresponding to $A^{-1}$ to the h^2^ corresponding to $G^{-1}$. In response to $A^{-1}$ weighted by α, $G^{-1}-A_{22}^{-1}$ in Equation (6) should be changed to $G^{-1}-\alpha A_{22}^{-1}$, which is equivalent to multiplying $\left[\begin{array}{cc} A^{11} & A^{12}\\ A^{21} & A^{22}-A_{22}^{-1} \end{array}\right]$ in Equation (11) by α. With an h^2^ estimate based on pedigree information, weighting $G^{-1}$ is preferred over weighting $A^{-1}$.

### 2.6. Weighting $A^{11}$

Considering Equation (10), weighting $A^{11}$ is equivalent to weighting all the components of $H^{-1}$, except $G^{-1}$, similar to that of the weighting $A^{-1}$ scenario.

### 2.7. Weighting $A^{22}$

Considering Equation (11), weighting $A^{22}$ should coincide with weighting the other blocks of $A^{-1}$ to preserve its conditional properties, as well as weighting $A_{22}^{-1}$, similar to that of the weighting $A^{-1}$ scenario.

### 2.8. Weighting $A_{22}^{-1}$

Considering Equation (10), weighting $A_{22}^{-1}$ is equivalent to:

(12) $$\begin{array}{cc} H^{-1} & =\left[\begin{array}{c} I\\ -\sqrt{\alpha }A_{22}^{-1}A_{21} \end{array}\right]A^{11}\left[\begin{array}{c} I\\ -\sqrt{\alpha }A_{12}A_{22}^{-1} \end{array}\right]+\left[\begin{array}{cc} 0 & 0\\ 0 & G^{-1} \end{array}\right]\\ \; & =\left[\begin{array}{cc} A^{11} & \sqrt{\alpha }A^{12}\\ \sqrt{\alpha }A^{21} & \alpha \left(A^{22}-A_{22}^{-1}\right) \end{array}\right]+\left[\begin{array}{cc} 0 & 0\\ 0 & G^{-1} \end{array}\right]\\ \; & =\left[\begin{array}{cc} I & 0\\ 0 & \sqrt{\alpha }I \end{array}\right]\left[\begin{array}{cc} A^{11} & A^{12}\\ A^{21} & A^{22} \end{array}\right]\left[\begin{array}{cc} I & 0\\ 0 & \sqrt{\alpha }I \end{array}\right]+\left[\begin{array}{cc} 0 & 0\\ 0 & G^{-1}-\alpha A_{22}^{-1} \end{array}\right]. \end{array}$$

However, this is not recommended as it imposes a different pedigree-based h^2^ on the genotyped and non-genotyped animals in $A^{-1}$. Furthermore, as α becomes smaller, the relationships between genotyped and non-genotyped animals are weakened.

### 2.9. The Experiments

Since the scenarios of weighting $A^{11}$ and $A^{22}$ are equivalent to weighting $A^{-1}$, and weighting $A_{22}^{-1}$ is not recommended, the four scenarios of weighting $H^{-1}$, $G^{-1}-A_{22}^{-1}$, $G^{-1}$, and $A^{-1}$ were tested. These scenarios were tested with α ranging from 0.8 to 1.2 to know the responses of each $H^{-1}$ conversion to the deviation of α from 1. Because weighting $G^{-1}-A_{22}^{-1}$ requires α to be between 0 and 1, it was studied with α ranging from 0.8 to 1. Predictive ability was calculated as Pearson’s correlation between the phenotypes and the estimated breeding values. Phenotypes were regressed on the estimated breeding values, where a lower slope means inflation and a higher slope means deflation.

## 3. Materials

Data were simulated for a species in a 1:1 sex ratio, litter size of 2, and generation overlap of 1. The pedigree, phenotypes, and genotypes were simulated using the R package pedSimulate [21]. Initially, ten generations were simulated, starting with a base generation (F0) of 100 animals (50 of each sex). No non-random pre-mating mortality or selection was applied to F0. Genotypes were simulated on 5000 markers, and allele frequencies were sampled from a uniform distribution ranging from 0.1 to 0.9. Marker (allele substitution) effects were simulated from a gamma distribution with shape and rate parameters equal to 2. The distribution was rebased to have a mean of 0 and scaled to create a variance of (true) marker breeding values in F0, $\sigma_{g}^{2}$ = 9. Residual polygenic and environment (residual) effects were simulated from normal distributions with variances $\sigma_{a}^{2}$ = 1 and $\sigma_{e}^{2}$ = 30, respectively.

Following F0, half of the males were mated to half of the females, which were all randomly selected and mated. Where the numbers of mating animals per sex were not equal, the sex with the higher number of animals underwent random selection to match the number of animals of the opposite sex. These ten generations were followed by ten more generations, in which 50% of male candidates (to become sires of the next generation) were selected for their marker breeding value and mated to the same number of randomly selected females. Genotypes in each subsequent generation were obtained by combining sampled gametes from the parents’ genotypes.

Phenotypes were calculated as y=μ1+g+a+e, where μ is the population mean, and **g**, **a**, and **e** are the vectors of effects corresponding to $\sigma_{g}^{2}$, $\sigma_{a}^{2}$, and $\sigma_{e}^{2}$. Genotypes before F8 and phenotypes for the last generation (F19) and before F7 were set to missing. Randomly, 5% of the known dams and 5% of the known sires (after F0) were set to missing. As such, missing pedigree and phenotype information, genomic pre-selection, and base and scale deviations between **A** and **G** were accommodated in the simulation. Data simulation was repeated ten times to reduce the possibility of observing the results specific to a dataset.

No fixed effect was simulated, and the data were analysed using the following mixed model equations:

(13) $$\left[\begin{array}{cc} 1^{\prime }1 & 1^{\prime }Z\\ Z^{\prime }1 & Z^{\prime }Z+H^{-1}\frac{\sigma_{e}^{2}}{\sigma_{g}^{2}+\sigma_{a}^{2}} \end{array}\right]\left[\begin{array}{c} \hat{\mu }\\ \hat{u} \end{array}\right]=\left[\begin{array}{c} \sum y\\ Z^{\prime }y \end{array}\right],$$

where **Z** is the matrix relating phenotypes to animals, **1** and $\hat{u}$ are the vectors of ones and predicted breeding values, and $\hat{\mu }$ is the mean estimate. Matrix **G** was used in $H^{-1}$ and built according to method 1 of VanRaden [5], where $G={\mathrm{WW}}^{\prime }/2\sum p\left(1-p\right)$, **W** is the centred and scaled genotype matrix, and *p* is the marker allele frequency. Markers with minor allele frequency below 0.02 were discarded before calculating **G**. Then, **G** was blended as $G\leftarrow 0.9G+0.1A_{22}$.

## 4. Results

**Figure 1:**
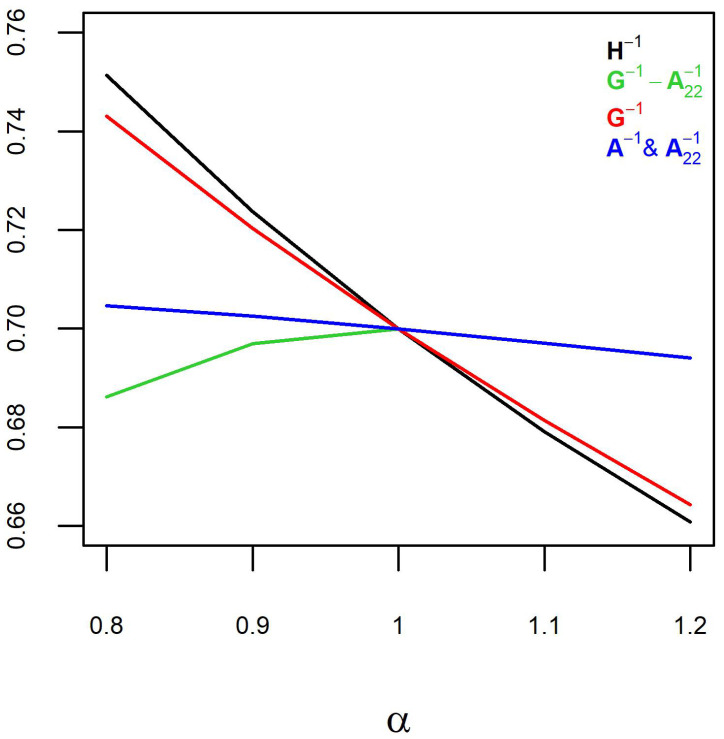
Regression coefficients of the phenotypes on genomic breeding values for different components of $H^{-1}$ weighted by α. Each data point is an average of ten observations for the simulated populations.

**Figure 2:**
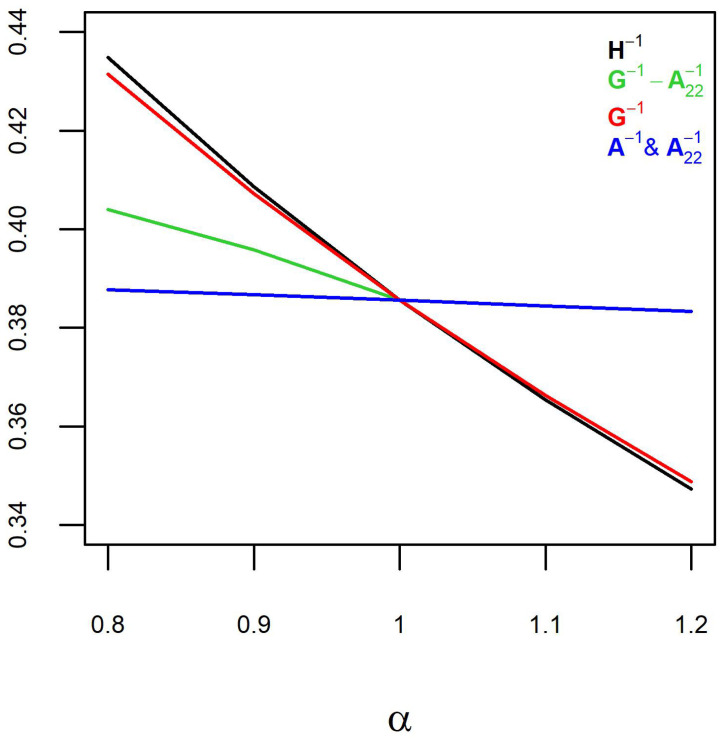
Correlation coefficients between phenotypes and genomic breeding values for different components of $H^{-1}$ weighted by α. Each data point is an average of ten observations for the simulated populations.

The simulated pedigrees had a population size of 2162.8 ± 358.3 (μ ± sd), 1326.4 ± 298.2 genotypes, 1324.6 ± 277.2 phenotypes, 1074.7 ± 156.8 males, and 1088.1 ± 202.9 females. Inflation and predictive ability estimates over the ten simulated pedigrees were averaged and presented (Figure 1 and Figure 2).

Different $H^{-1}$ components were weighted by α ranging from 0.8 to 1.2, except for $G^{-1}-A_{22}^{-1}$, where α ranged from 0.8 to 1. Weighting $H^{-1}$ and $G^{-1}$ showed similar trends for inflation (Figure 1) and predictive ability (Figure 2), with the slope of the trends being slightly less for $G^{-1}$ compared to $H^{-1}$. Weighting $A^{-1}$ (accompanied by weighting $A_{22}^{-1}$) showed slightly decreasing trends, with the regression slope decreasing by 0.01 (i.e., inflation increasing by 0.01) and the predictive ability decreasing by 4.4 $\times 10^{-3}$ over the range of α. The inflation and prediction ability increased by weighting $G^{-1}-A_{22}^{-1}$ with α decreasing from 1 to 0.8.

## 5. Discussion

Matrices **G** and $A_{22}$ indicate different means and variances for genotyped animals. This can cause differently scaled genomic and pedigree information in $H^{-1}$ [3]. Usually, **G** is blended and tuned (rebased and scaled) with $A_{22}$. If genomic breeding values are still inflated, a complementary weighting of $G^{-1}-A_{22}^{-1}$ might be needed. A common practice is to weight using $\tau G^{-1}-\omega A_{22}^{-1}$. It was shown that some τ≠ω combinations are likely to distort the properties of **H** that provide conditionality between the breeding values of genotyped and non-genotyped animals. Other ways of weighting the components of $H^{-1}$ were presented that are unlikely to distort the conditional properties of **H**.

Weighting $H^{-1}$ with α > 1 is equivalent to reducing h^2^ and increasing inflation due to increased dispersion. It is equivalent to adding (1−α)/α to 1/h^2^ or weighting the genetic variance by 1/α. Due to selection, h^2^ can be lower than expected. The h^2^ reduction is expected to be greater due to genomic selection. Change of genetic variance by genomic selection is propagated from **G** throughout **H**. The predictive ability declined with increasing α (Figure 2), which might be concerning. However, predictive ability is a direct function of the slope of the regression line (Figure 1). Therefore, the slope of the regression line (inflation) should be the main concern.

Weighting $A^{-1}$ (accompanied by weighting $A_{22}^{-1}$) did not influence inflation and predictive ability. Predictive ability and the slope of the regression line decreased slightly (inflation increased slightly) over the increase in α. The reason for this is likely that **H** is a genomic relationship matrix extended from **G** for genotyped animals to non-genotyped animals via the $A_{12}A_{22}^{-1}$ coefficients (Equations (2)–(5)). As such, **G** is more influential in defining the variances in **H** than **A**. This was confirmed by similar trends for weighting $G^{-1}$ and $H^{-1}$ (Figure 1 and Figure 2). The slopes of the regression line (inflation) and predictive ability were slightly steeper for $H^{-1}$ than for $G^{-1}$, and that was a result of the combined weighting of $G^{-1}$, $A^{-1}$ and $A_{22}^{-1}$. Weighting $G^{-1}-A_{22}^{-1}$ by α < 1 increased the inflation but at a lower rate than weighting $H^{-1}$ or $G^{-1}$ with α > 1.

The inflation results are expected to be valid for other data as weighting $H^{-1}$ or its components is equivalent to inversely weighting the genetic variance, regardless of the data. The exception is weighting $G^{-1}-A_{22}^{-1}$. Whether weighting $G^{-1}-A_{22}^{-1}$ with a larger α results in inflation or deflation depends on whether using $H^{-1}$ instead of $A^{-1}$ results in inflation or deflation. If using $H^{-1}$ results in inflation, then weighting $G^{-1}-A_{22}^{-1}$ with a larger α (more emphasis on $H^{-1}$ than $A^{-1}$) results in greater inflation. The predictive ability improved by weighting $G^{-1}-A_{22}^{-1}$ with α decreasing from 1 to 0.8. Generally, predictive ability increases by the increase in the slope of the regression line. Notice that the predictive ability ignoring inflation can be misleading. Since the trends for prediction ability and the slope of the regression line were in opposite directions for weighting $G^{-1}-A_{22}^{-1}$, it shows that the predictive ability benefited from blending $H^{-1}$ and $A^{-1}$, mainly because the h^2^ was more compatible with a blended $H^{-1}$ and $A^{-1}$ than with $H^{-1}$.

This study does not completely rule out using $\tau G^{-1}-\omega A_{22}^{-1}$. However, weighting $H^{-1}$ components should meet specific conditions to avoid/minimise violating the conditional properties of **H**. As such,

$$\begin{array}{c} A^{-1}+\left[\begin{array}{cc} 0 & \;0\\ 0 & \alpha \left(G^{-1}-A_{22}^{-1}\right) \end{array}\right],\\ \tau \left(A^{-1}+\left[\begin{array}{cc} 0 & \;0\\ 0 & \alpha \left(G^{-1}-A_{22}^{-1}\right) \end{array}\right]\right),\\ A^{-1}+\left[\begin{array}{cc} 0 & \;0\\ 0 & \alpha G^{-1}-A_{22}^{-1} \end{array}\right], \end{array}$$

and $\alpha H^{-1}$ are better alternatives to $\tau G^{-1}-\omega A_{22}^{-1}$. By definition, none of these four options are better than the others. However, achieving good compatibility between the resulting $H^{-1}$ and h^2^ without blending $H^{-1}$ and $A^{-1}$ at a high rate (low emphasis on genomic information) is important.

Concerning pedigree and genomic errors, regardless of the emphasis given to pedigree and genomic information, genotype errors propagate through non-genotyped animals, and pedigree errors incorrectly and insufficiently propagate genotype information through non-genotyped animals. Therefore, the correctness and the completeness of pedigree and genomic information are vital for accurate and unbiased ssGBLUP evaluations.

Future research may focus on changing genetic parameters over time or across populations in genomic predictions. It is possible to reduce inflation in genomic predictions for young animals by using smaller additive genetic variances. This can be done by replacing $H^{-1}$ with ${\mathrm{DH}}^{-1}D$. Considering no overall weight on $H^{-1}$: $\sum {\mathrm{DH}}^{-1}D=\sum H^{-1}$. Matrix **D** is a diagonal matrix of positive values descending in function of the animal’s age. The researcher would need to decide the $\min\left(d\right)\leq \frac{\sigma_{e}}{\sigma_{g}}\leq \max\left(d\right)$ range, where **d** = diag(**D**). With recent advances in ssGBLUP (mentioned by Misztal et al. [18]), which improve the compatibility between **A** and **G**, conditioning $H^{-1}$ might become an interim solution from the past or be reduced to only weighting $H^{-1}$.

## Data Availability

The data, code, and the results supporting the findings of this study are openly available in Mendeley Data at doi:10.17632/cn9jzpj7fg.1 [22].

## References

[1] Misztal I., Legarra A., Aguilar I. (2009). Computing procedures for genetic evaluation including phenotypic, full pedigree, and genomic information. J. Dairy Sci..

[2] Legarra A., Aguilar I., Misztal I. (2009). A relationship matrix including full pedigree and genomic information. J. Dairy Sci..

[3] Aguilar I., Misztal I., Johnson D.L., Legarra A., Tsuruta S., Lawlor T.J. (2010). Hot topic: A unified approach to utilise phenotypic, full pedigree, and genomic information for genetic evaluation of Holstein final score. J. Dairy Sci..

[4] Christensen O.F., Lund M.S. (2010). Genomic prediction when some animals are not genotyped. Genet. Sel. Evol..

[5] VanRaden P.M. (2008). Efficient methods to compute genomic predictions. J. Dairy Sci..

[6] Gao H., Christensen O.F., Madsen P., Nielsen U.S., Zhang Y., Lund M.S., Su G. (2012). Comparison on genomic predictions using three GBLUP methods and two single-step blending methods in the Nordic Holstein population. Genet. Sel. Evol..

[7] Forni S., Aguilar I., Misztal I. (2011). Different genomic relationship matrices for single-step analysis using phenotypic, pedigree and genomic information. Genet. Sel. Evol..

[8] Christensen O.F. (2012). Compatibility of pedigree-based and marker-based relationship matrices for single-step genetic evaluation. Genet. Sel. Evol..

[9] Nilforooshan M.A. (2020). Application of single-step GBLUP in New Zealand Romney sheep. Anim. Prod. Sci..

[10] Vitezica Z.G., Aguilar I., Misztal I., Legarra A. (2011). Bias in genomic predictions for populations under selection. Genet. Res..

[11] Chen C.Y., Misztal I., Aguilar I., Legarra A., Muir W.M. (2011). Effect of different genomic relationship matrices on accuracy and scale. J. Anim. Sci..

[12] Martini J.W.R., Schrauf M.F., Garcia-Baccino C.A., Pimentel E.C.G., Munilla S., Rogberg-Muñoz A., Cantet R.J.C., Reimer C., Gao N., Wimmer V. (2018). The effect of the **H**^−1^ scaling factors *τ* and *ω* on the structure of **H** in the single-step procedure. Genet. Sel. Evol..

[13] Misztal I., Aguilar I., Legarra A., Lawlor T.J. Choice of parameters for single-step genomic evaluation for type. Proceedings of the 61st Annual EAAP Meeting.

[14] Kang H., Ning C., Zhou L., Zhang S., Yan Q., Liu J.-F. (2018). Short communication: Single-step genomic evaluation of milk production traits using multiple-trait random regression model in Chinese Holsteins. J. Dairy Sci..

[15] Imai A., Kuniga T., Yoshioka T., Nonaka K., Mitani N., Fukamachi H., Hiehata N., Yamamoto M., Hayashiet T. (2019). Single-step genomic prediction of fruit-quality traits using phenotypic records of non-genotyped relatives in citrus. PLoS ONE.

[16] Alvarenga A.B., Veroneze R., Oliveira H.R., Marques D.B.D., Lopes P.S., Silva F.F., Brito L.F. (2020). Comparing alternative single-step GBLUP approaches and training population designs for genomic evaluation of crossbred animals. Front. Genet..

[17] Fu C., Ostersen T., Christensen O.F., Xiang T. (2021). Single-step genomic evaluation with metafounders for feed conversion ratio and average daily gain in Danish Landrace and Yorkshire pigs. Genet. Sel. Evol..

[18] Misztal I., Lourenco D., Tsuruta S., Aguilar I., Masuda Y., Bermann M., Cesarani A., Legarra A. How ssGBLUP became suitable for national dairy cattle evaluations. Proceedings of the 12th World Congress on Genetics Applied to Livestock Production.

[19] Lourenco D.A.L., Legarra A., Tsuruta S., Masuda Y., Aguilar I., Misztal I. (2020). Single-step genomic evaluations from theory to practice: Using SNP chips and sequence data in BLUPF90. Genes.

[20] Legarra A. (2016). Comparing estimates of genetic variance across different relationship models. Theor. Pop. Biol..

[21] Nilforooshan M.A. (2022). pedSimulate—An R package for simulating pedigree, genetic merit, phenotype, and genotype data. R. Bras. Zootec..

[22] Nilforooshan M.A. Code & Data—A Note on the Conditioning of the H-1 Matrix Used in Single-Step GBLUP. Mendeley Data V1. 2022.

